# Microsurgical management of midbrain cavernous malformations: does lesion depth influence the outcome?

**DOI:** 10.1007/s00701-021-04915-y

**Published:** 2021-08-20

**Authors:** Caiquan Huang, Helmut Bertalanffy, Souvik Kar, Yoshihito Tsuji

**Affiliations:** 1 Department of Neurosurgery, International Neuroscience Institute (INI), Rudolf Pichlmayr-Strasse 4, 30625 Hannover, Germany; 2 Department of Neurosurgery, Sichuan Provincial People’s Hospital, University of Electronic Science and Technology of China, Chengdu, China; 3 Department of Neurosurgery, Matsubara Tokushukai Hospital, Matsubara, Japan

**Keywords:** Brainstem cavernous malformation, Midbrain, Indication for surgery, Surgical approach, Surgical technique, Vascular disorders

## Abstract

**Background:**

The purpose of this study was to clarify whether the intrinsic depth of midbrain cavernous malformations (MCMs) influenced the surgical outcome.

**Methods:**

The authors conducted a retrospective study of 76 consecutive patients who underwent microsurgical resection of a MCM. The vascular lesions were categorized into 4 distinct groups based on how these lesions had altered the brainstem surface. Additionally, it was verified whether the actual aspect of the brainstem surface could be predicted only by evaluating the pertinent preoperative MRI slices. Clinical outcome was assessed by determining the modified Rankin Scale Score (mRS) before and after surgery.

**Results:**

Twenty-three MCMs (30.3%) were located deeply within the midbrain. The overlying midbrain surface appeared to be normal (group *nl*). In 33 patients (43.4%), the midbrain surface showed only a yellowish discoloration (group *yw*). In another 14 individuals (18.4%), the midbrain surface was distorted by the underlying MCM and bulging out while the vascular lesion still remained covered by a thin parenchymal layer (group *bg*). In the smallest group comprising 6 patients (7.9%), the exophytic MCM had disrupted the midbrain surface and was clearly visible at microsurgical exposure (group *ex*). The mean mRS decreased in the group nl from 1.43 preoperatively to 0.61 at follow-up.

**Conclusion:**

This study demonstrates in a large patient population that a deep intrinsic MCM location is not necessarily associated with an unfavorable clinical outcome after microsurgical lesionectomy. Predicting the aspect of the midbrain surface by evaluating preoperative MR images alone was not sufficiently reliable.

## Introduction

In our previous clinical study of midbrain cavernous malformations (MCMs), we presented a lesion classification and identified predictors of surgical outcome [29]. However, the impact of MCM depth on the surgical outcome, particularly in lesions that have not visibly altered the surface of the midbrain, has not yet been assessed. In the pertinent literature, such lesions are generally not considered good candidates for microsurgical resection [1, 10, 26, 32]. To shed more light onto this important matter, we analyzed the relationship and measured the distance between intrinsic MCMs and midbrain surface in our surgical case series. Our main focus lay on correlating the intra-axial depth of MCM location with the surgical outcome.

## Patients and methods

We retrospectively analyzed clinical and surgical data including video records of each surgical procedure, neuroradiological findings, and follow-up results of 76 consecutive patients who underwent microsurgical removal of a MCM. These patients form a subgroup of 302 consecutive individuals who underwent microsurgical removal of a brainstem CM during the past 25 years. All patients were surgically treated between 1996 and 2021 by the senior author (HB) mainly at three different institutions.

Patients were selected as surgical candidates when they suffered from clinically pertinent or repeated intrinsic mesencephalic hemorrhage associated with mass effect, regardless of whether the lesion had reached the pial or ependymal brainstem surface. All patients underwent preoperatively a routine MRI examination. Lesion size (largest diameter) as well as distance between MCM and midbrain surface expressed in millimeters were determined on high-resolution T1- and T2-weighted preoperative and postoperative MRI slices, either in the axial or in the sagittal plane.

The MCMs in this series were exposed via several surgical access routes that are listed in the "Results" section. Generally, the area where the underlying MCM appeared to be closest to the midbrain surface was chosen as the surgical entry point into the brainstem.

During surgery, motor, sensory, and brainstem auditory–evoked potentials were applied in all individuals. In selected cases, we also used electromyography of cranial nerves III and IV. All patients underwent a control MRI examination within the first 48 h after surgery.

In addition to measurements on MRI slices, we also assessed the aspect of the midbrain surface in the area of the underlying MCM. Moreover, we documented the exact entry point into the brainstem and measured the width of the final midbrain aperture by reviewing relevant sections of each patient’s surgical video record. The operating surgeon’s habit to use a millimeter scale during the intradural procedure facilitated these retrospective measurements.

By surveying each patient’s midbrain surface on the respective video clip, we found 4 distinct morphological appearances:

1. The midbrain surface appears to be normal; there is no discoloration and no parenchymal bulge;
2. The midbrain surface shows only a yellowish discoloration caused by intraparenchymal hemosiderin deposits; no bulge is visible;
3. The midbrain surface is displaced by the intrinsic MCM and clearly bulges out; however, the pial surface is intact and overlies the MCM that is not directly visible; hemosiderin discoloration may be present.
4. The midbrain pial surface is disrupted by the MCM that partially bulges out of the brainstem; the lesion’s exophytic portions are not covered by a parenchymal layer and are directly visible in this area.

**Fig. 1:**
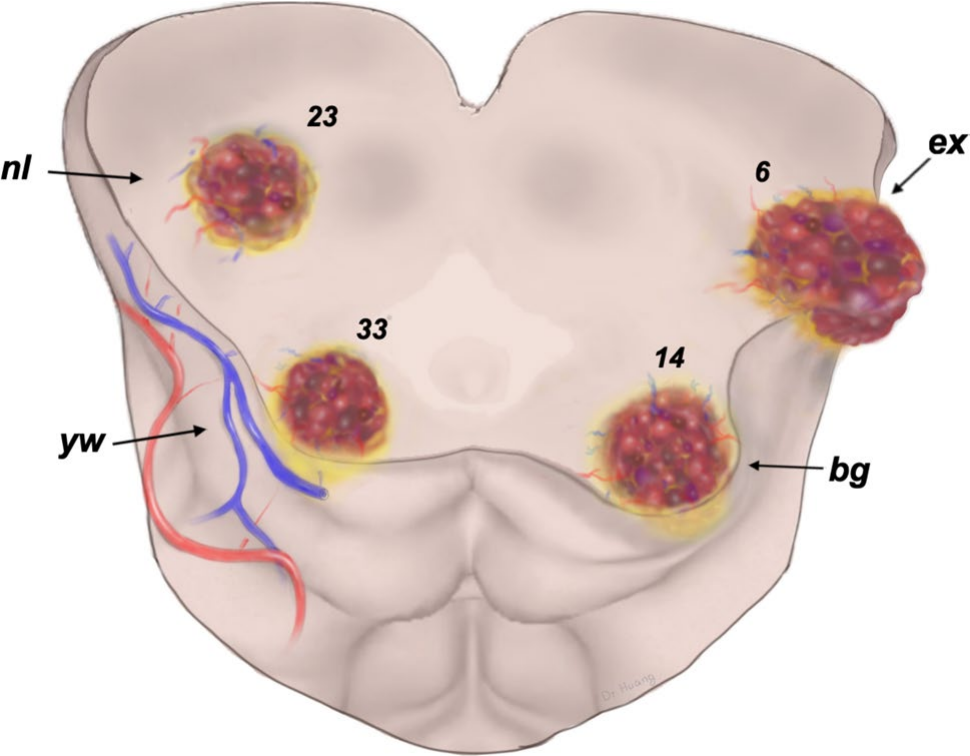
Artistic illustration of a section through the midbrain that contains the 4 MCM types and shows the relationship between lesion and brainstem surface. The numerals indicate the number of lesions in the present series. *nl*: the intrinsic MCM has not altered the midbrain surface, which appears to be normal; *yw*: the underlying MCM has produced only a yellowish discoloration of the midbrain surface by parenchymal hemosiderin deposits; *bg*: the intrinsic MCM is covered by a thin layer of parenchyma but causes superficial discoloration and a bulge of the midbrain surface; *ex*: the exophytic MCM has disrupted the pial midbrain surface and bulges out of the brainstem

**Fig. 2:**
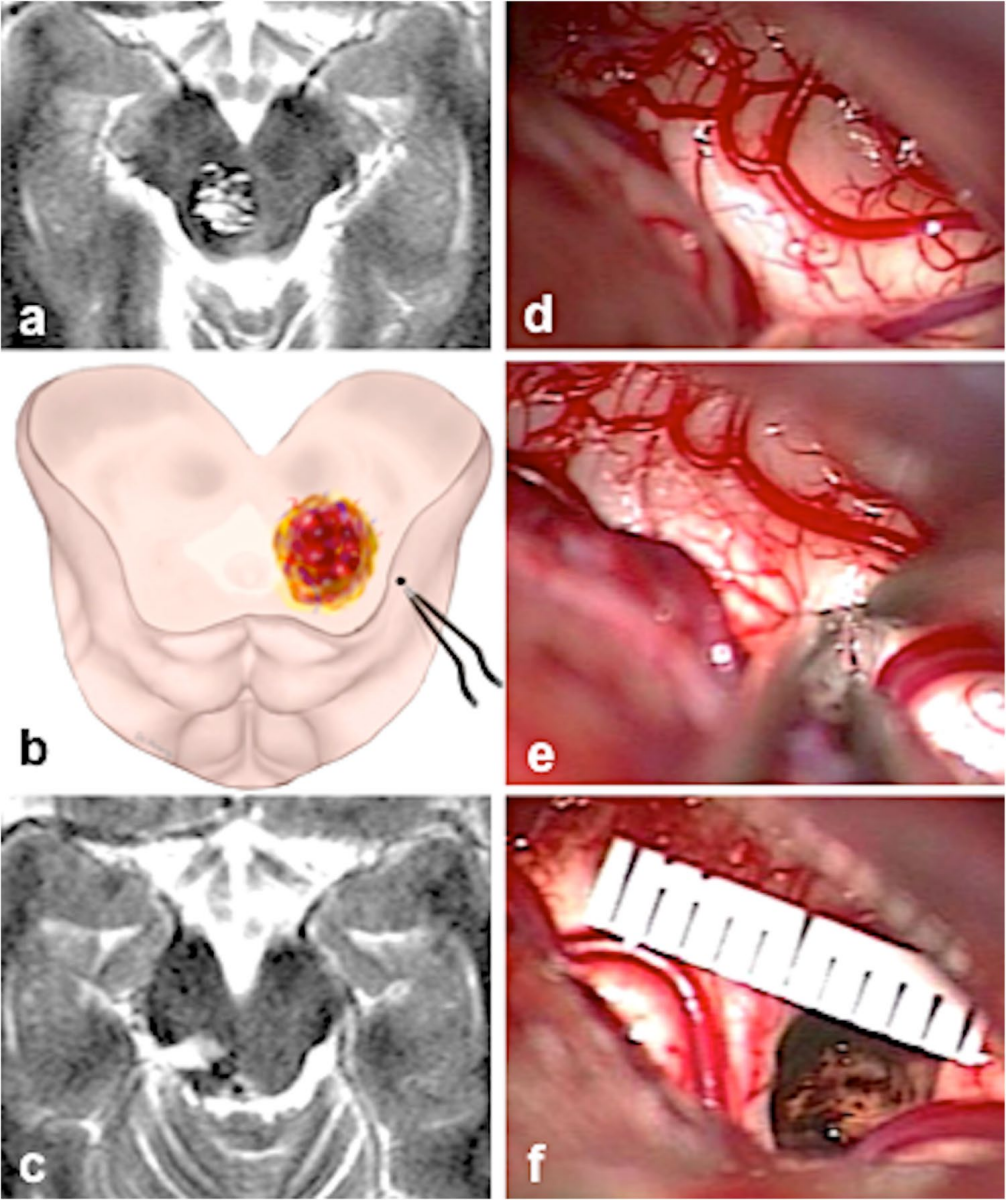
**a** Typical example of category *nl*; axial MRI with a deep-seated MCM. **b** Illustration showing the entry point into the brainstem behind the lateral mesencephalic sulcus. **c** Postoperative axial MRI with the resection cavity. **d** The right lateral surface of the midbrain appeared normal at surgery. **e** The entry point into the midbrain. **f** The resection cavity with a millimeter scale

**Fig. 3:**
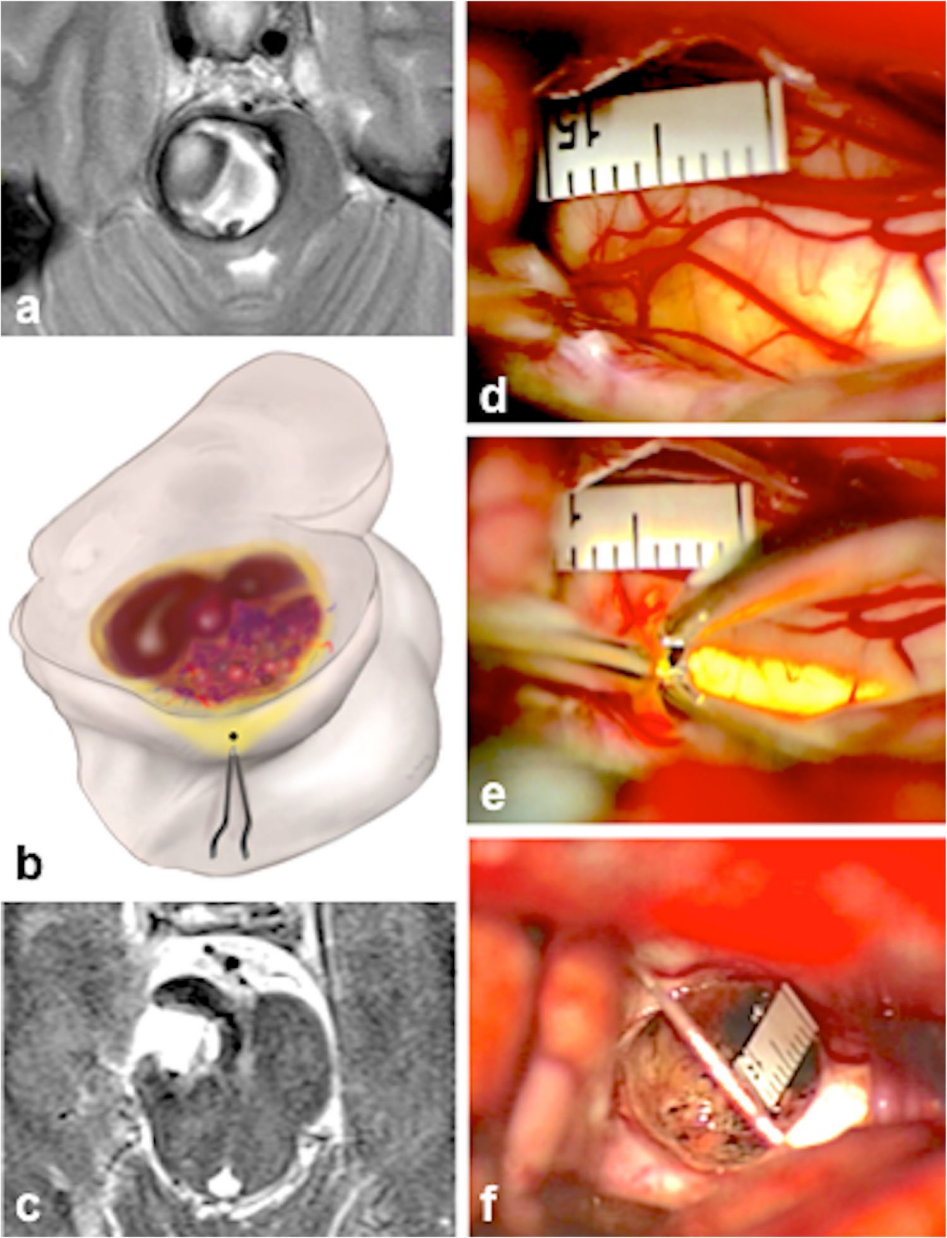
Typical example of the category *yw*. **a** Axial MRI with a deep-seated hemorrhagic MCM. **b** Illustration showing the entry point into the brainstem at the level of the lateral mesencephalic sulcus. **c** Postoperative axial MRI with the resection cavity. **d** The right lateral surface of the midbrain peduncle shows a yellowish discoloration at surgery. **e** The entry point into the midbrain peduncle. **f** The resection cavity with a millimeter scale

**Fig. 4:**
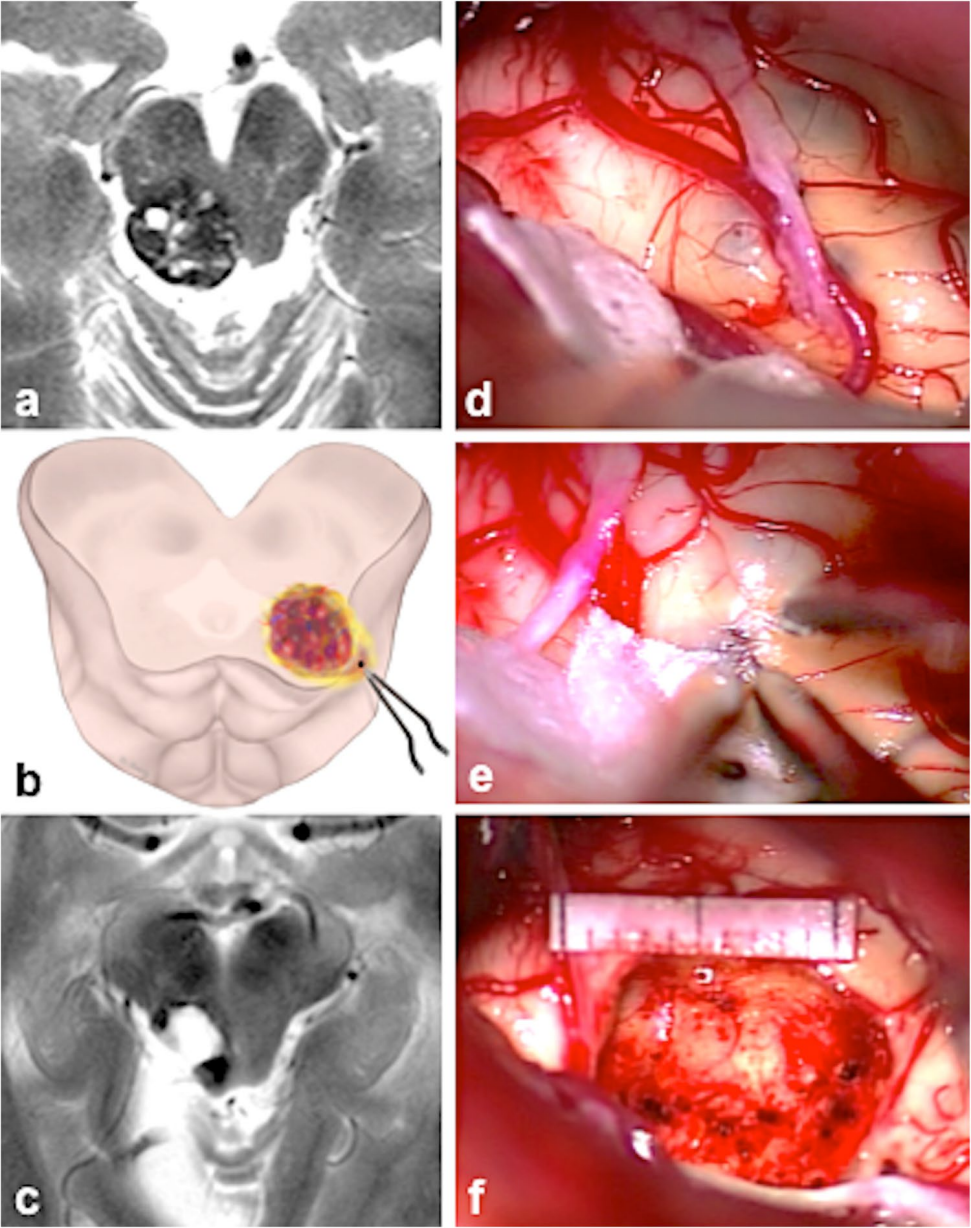
Typical example of the category *bg*. **a** Axial MRI with a superficial tectal/tegmental MCM. **b** Illustration showing the entry point into the right lateral tectum. **c** Postoperative axial MRI with the resection cavity. **d** The right lateral surface of the midbrain is prominent at surgery, the underlying MCM shines through the pial surface. **e** The entry point into the midbrain. **f** The resection cavity with a millimeter scale

**Fig. 5:**
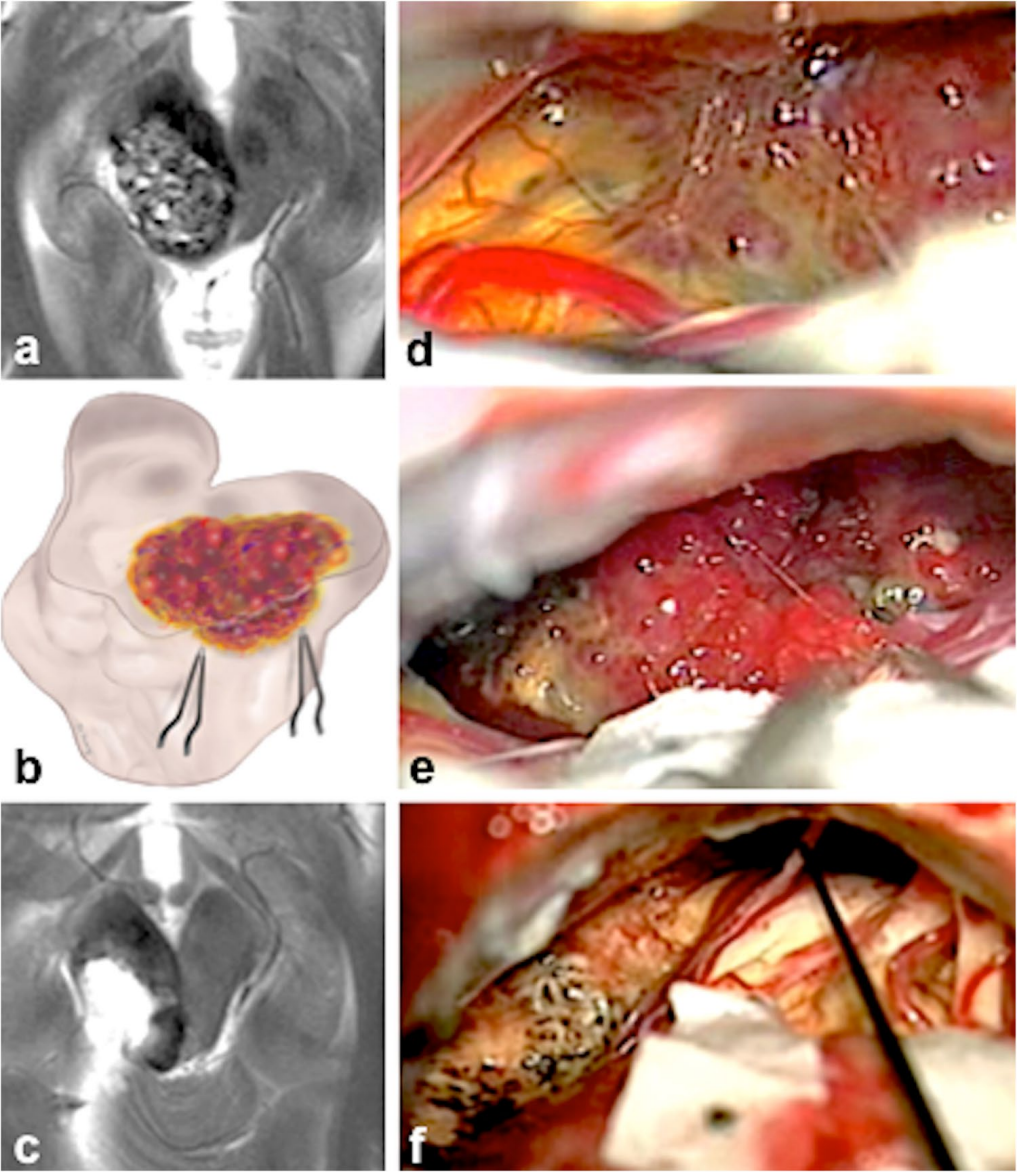
Typical example of the category *ex*. **a** Axial MRI with a superficial tegmental/tectal MCM. **b** Illustration showing the exophytic MCM. **c** Postoperative axial MRI with the resection cavity. **d** Intraoperatively, the MCM is readily visible on the right tegmental surface. **e** The lesion is gradually exposed. **f** The resection cavity and cranial nerves IV, V, and VII

Based on these definition criteria, and as shown in Figs. 1, 2, 3, 4, and 5, we divided all MCMs into 4 distinct types termed as follows: *nl* (for normal midbrain aspect), *yw* (for yellow discoloration), *bg* (for bulging surface), and *ex* (for exophytic lesion). Correspondingly, we distinguished 4 patient groups that harbored one of these 4 lesion types.

In a separate step, we analyzed each patient’s pertinent MRI slices and allocated them to one of the categories (*nl*, *yw*, *bg*, and *ex)* by estimating the most likely aspect of the midbrain surface in the area of the underlying MCM as suggested by the respective MRI appearance. We undertook this initial MRI evaluation in a blinded fashion without being aware of the intraoperative finding. In the following step, we compared these assumptive appearances derived from examining only the MRI slices with the actual aspect of the brainstem surface as reviewed on the respective video records.

To assess the surgical outcome, we determined the modified Rankin Scale Score (mRS) before and after the operation and documented oculomotor deficits.

Follow-up evaluation was obtained by examining the patients during outpatient visits at our hospital and, in a few cases, by telephone or e-mail questionnaires. At least 2 physicians undertook these postoperative evaluations (one treating neurosurgeon and another doctor who was not directly involved in the surgical treatment). Yet, in a few further cases, foreign physicians provided us detailed follow-up data of our previous patients from abroad.

### Statistical analysis

Data analysis was performed using IBM SPSS (version 25.0, IBM Corp.) on a personal computer. Baseline characteristics were analyzed as mean ± SD or as median and IQRs as appropriate. We used independent-samples *t* test, Fisher’s exact test, the Wilcoxon-Mann–Whitney test, as well as the one-way-ANOVA and Kruskal–Wallis test. A *P* value < 0.05 was considered statistically significant. Cohen’s kappa was used to assess the congruence between the aspect of the midbrain surface as derived from preoperative MRI slices and the superficial appearance as observed on surgical video clips.

## Results

### Patient and lesion characteristics

All 76 patients (100%) had a previous history of hemorrhage with at least one bleeding episode. Baseline demographic and clinical variables in the entire patient population and in the 4 groups (*nl*, *yw*, *bg*, and *ex*) are listed in Table 1. Gender, number of preoperative hemorrhagic events, time interval from the last bleeding episode to surgery (here 6 weeks), and poor mRS on admission did not differ significantly among the groups. However, a significantly smaller lesion size was observed in the groups *nl* and *yw* compared to the groups *bg* and *ex*. The largest MCMs were found in the group *bg*.

**Table 1:** Patient and lesion characteristics

	Total	*nl*	*yw*	*bg*	*ex*	*P* value
Distribution, *N* (%)	76 (100)	23 (30.3)	33 (43.4)	14 (18.4)	6 (7.9)	
Age (mean ± SD, range), years	35.4 ± 13.2, 1–70	33.1 ± 11.9, 16–61	40.2 ± 11.6, 19–70	31.5 ± 12.3, 14–58	27 ± 20.8, 1–55 *	0.049 ^a^
Male sex, *N* (%)	36 (47.3)	14 (60.9)	15 (45.5)	5 (35.7)	2 (33.3)	0.425 ^b^
Lesion size (mean ± SD, range), mm	19.0 ± 8.2, 4–55	17.4 ± 7.2, 7–36	15.9 ± 6.5, 4–30	26.2 ± 9.1, 17–55 *	24.8 ± 6.8, 20–35 *	< 0.001 ^c^
No. of preoperative hemorrhagic events (mean ± SD)	1.7 ± 0.8	1.52 ± 0.68	1.83 ± 0.73	2 ± 0.96	2 ± 1.22	0.217 ^c^
Resection within 6 weeks	23/76, 30.3%	6/23, 26.1%	9/33, 27.3%	7/14, 50%	1/6, 16.7%	0.329 ^b^
Poor mRS (3–5) on admission	10/76, 13.2%	1/23, 4.3%	4/33, 12.1%	3/14, 21.4%	2/6, 33.3%	0.144 ^b^
Distance between MCM and midbrain surface (median, range, IQR), mm	2, 1–10, 1–3	3, 1–10, 2–4	2, 1–3, 1–2	1, 1–2, 1–1.125	–	< 0.001 ^c^
Width of midbrain aperture (median, range, IQR), mm	6, 3–16, 5–8.25	5, 4–11, 5–7	6, 3–13, 5–8.5	7, 4–16, 6–9	5, 5–12, 5.5–10.5	0.32 ^c^

^a^One-way ANOVA

^b^Fisher’s exact test

^c^Kruskal-Wallis test

^*^Significantly different (*P* < 0.05) compared to the group *nl*

### Surgical approaches, entry points into the brainstem, and extent of resection

**Fig. 6:**
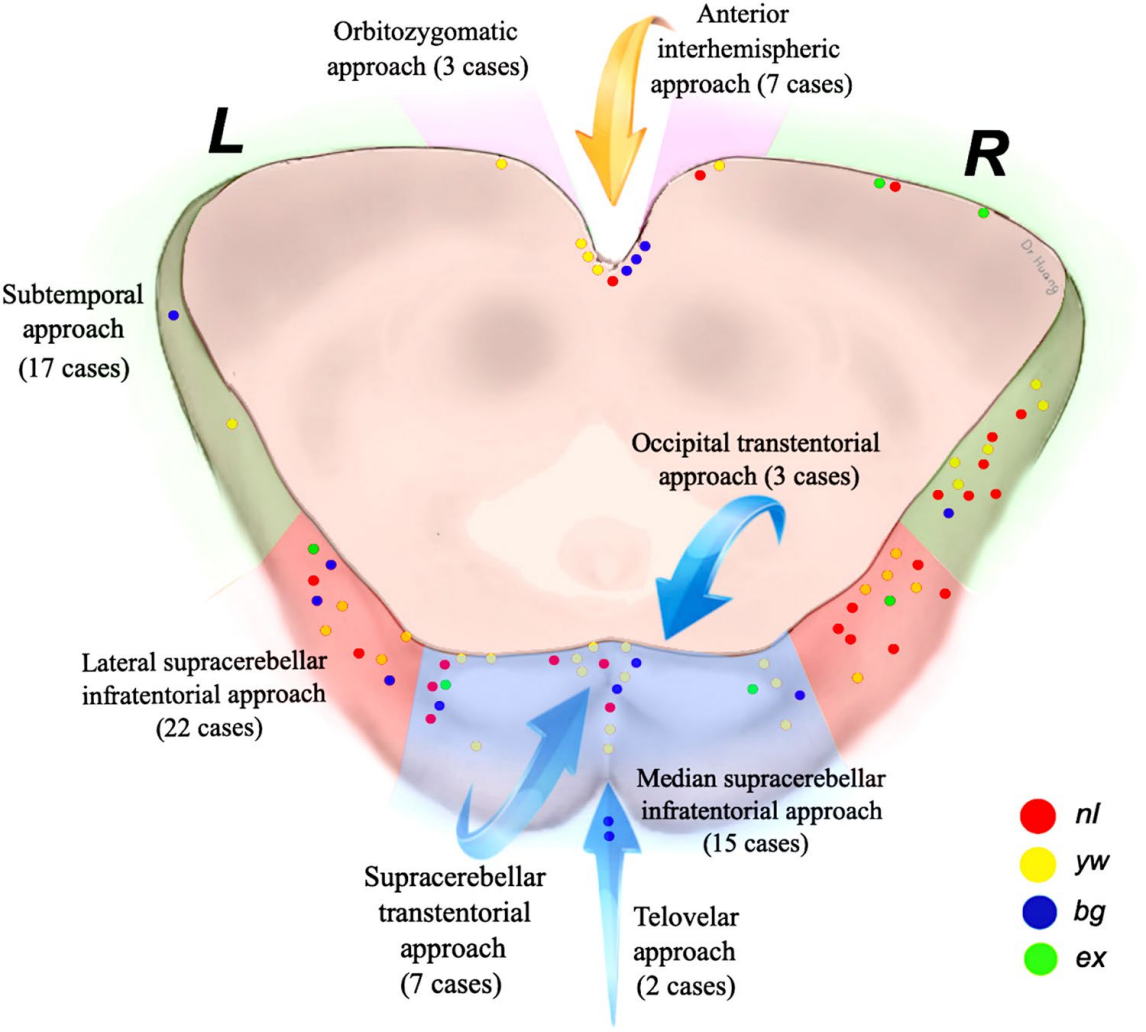
Artistic illustration demonstrating the area of midbrain exposure obtained by the various surgical approaches used in this series. Each dot indicates the approximate center of the surgical midbrain aperture that was used as the entry point into the brainstem. The dot colors correspond to one of the 4 lesion types as shown in the color legend. The right-sided subtemporal approach was used more frequently to access the MCMs to avoid the risk of injuring the dominant temporal lobe

We used 8 different surgical approaches to access the MCMs in this series as listed in Table 2, which also shows the distribution among the patient groups. Figure 6 illustrates the area of midbrain exposure according to the various surgical approaches and depicts each entry point on the midbrain surface. Understandably, the distance between MCM and midbrain surface was measured only in the groups *nl*, *yw*, and *bg*. In the group *bg*, this distance corresponded to the thickness of the superficial parenchymal layer. All values as well as the width of the aperture at the entry point into the midbrain are listed in Table 1. In contrast to the width of midbrain aperture that showed only minimal differences among patient groups, and as a matter of course, the distance between MCM and midbrain surface varied significantly among the 3 groups (*nl*, *yw*, and *bg*). The deepest MCM location (10 mm from the midbrain surface) was found in the group *nl*, while the largest midbrain aperture (16 mm) was observed in the group *bg*. Complete MCM resection was achieved in all patients except for one individual in whom 1–2% of the MCM were deliberately left in place to avoid excessive manipulation within the vulnerable midbrain parenchyma.

**Table 2:** Surgical approach

	Total	*nl*	*yw*	*bg*	*ex*
Anterior interhemispheric approach	7	1	3	3	0
Orbitozygomatic approach	3	1	2	0	0
Subtemporal approach	17	7	6	2	2
Lateral supracerebellar infratentorial approach	22	8	9	3	2
Median supracerebellar infratentorial approach	15	4	8	2	1
Supracerebellar transtentorial approach	7	1	4	2	0
Occipital transtentorial approach	3	1	1	0	1
Telovelar approach	2	0	0	2	0

### Surgical outcome

Regrettably, two patients were lost for long-term follow-up and were excluded from this analysis. In the remaining 74 patients, the long-term follow-up ranged from 6 months to 22 years, with a mean of 3.5 years. One male patient who was in excellent clinical condition after MCM removal died 4 years after surgery from a disease unrelated to the brainstem cavernoma; notwithstanding, his clinical condition was evaluated until shortly before his death. Forty-eight of 74 patients (64.9%) had an improved neurological condition at last follow-up. Table 3 shows the mean mRS on admission and at follow-up in the entire population as well as in each patient group. Obviously, the mean mRS at follow-up in the group *nl* was superior to the score in the groups *bg* and *ex* and in the entire patient population. In the 29 patients who presented with oculomotor nerve deficits at admission, 10 individuals had improved (34.5%) while the remaining 19 patients were found to have unchanged or slightly worsened third nerve deficits (65.5%); this difference, however, was statistically not significant (Table 4). There was no surgical mortality in this patient series.

**Table 3:** Preoperative and postoperative mRS

Patients; groups	*N*	Admission (mean ± SD)	Follow-up (mean ± SD)	*P* value ^a^
Total	74	1.55 ± 0.99	0.75 ± 0.98 *	< 0.001
*nl*	23	1.43 ± 0.66	0.61 ± 0.89 *	< 0.001
*yw*	31	1.55 ± 0.94	0.55 ± 0.75 *	< 0.001
*bg*	14	1.57 ± 1.5	1.14 ± 1.23	0.329
*ex*	6	2.00 ± 0.89	1.16 ± 1.17	0.132

^a^Wilcoxon-Mann–Whitney test

^*^Statistically significant improvement (*P* < 0.05) compared to preoperative mRS

**Table 4:** Preoperative and postoperative CN III nerve deficit

	CN III nerve deficit (on admission)	Improved CN III nerve deficit at follow-up	CN III nerve deficit same or worse at follow-up	*P* value
Total	29/74 (39.2%)	10/29 (34.5%)	19/29 (65.5%)	0.054 ^a^
*nl*	11/23 (47.8%)	5	6	
*yw*	10/21 (47.6%)	3	7	
*bg*	6/14 (42.9%)	0	6	
*ex*	2/6 (33.3%)	2	0	

^a^Fisher’s exact test

### Predictors of surgical outcome in the group nl

Because the superficial aspect of the midbrain was apparently normal in the 23 individuals of the group *nl*, access to the intrinsic MCM required splitting healthy midbrain parenchyma. For this reason we endeavored to identify predictors of outcome in this special patient group. As shown in Fig. 6, these MCMs were accessed surgically from all directions, most frequently, however, via the subtemporal approach (in 7 individuals, 26%) and via the supracerebellar infratentorial approach (in 14 patients, 61%). Table 5 lists a number of parameters that were analyzed to assess possible predictors of outcome in the group *nl* (patient’s age, gender, preoperative mRS < 3, number of preoperative hemorrhagic events, resection within 6 weeks, lesion size, distance between MCM and midbrain surface, and width of the midbrain aperture). Obviously, none of the examined parameters was identified as a reliable predictor. Two measurements were considered particularly important. However, even when analyzing differently as shown in Table 6, neither the distance between deep-seated MCM and midbrain surface nor the width of midbrain aperture influenced the surgical outcome significantly.

**Table 5:** Demographic, clinical, and intraoperative parameters of the group *nl* related to the outcome

Parameters	Improved mRS at follow-up	mRS same or worse at follow-up	*P* value
Patients, *n*/*N* (%)	15/23, 65.2%	7/23, 30.4%; 1/23, 4.3%	
Age (mean ± SD), years	33.13 ± 11.04	33.12 ± 14.1	0.796 ^a^
Male, *n*/*N* (%)	7/15, 46.7%	7/8, 87.5%	0.086 ^c^
Preoperative good mRS (0–2), *n*/*N* (%)	15/15, 100%	7/8, 87.5%	0.348 ^c^
Preoperative hemorrhagic events (mean ± SD)	1.46 ± 0.74	1.50 ± 0.53	0.912 ^b^
Resection within 6 weeks	5/15, 33.3%	1/8, 12.5%	0.369 ^c^
Lesion size (mean ± SD), mm	16.60 ± 7.47	18.80 ± 6.72	0.45 ^b^
Distance between MCM and midbrain surface (mean ± SD), mm	3.80 ± 2.21	2.62 ± 1.06	0.175 ^b^
Width of midbrain aperture (mean ± SD), mm	5.53 ± 1.68	7.00 ± 3.02	0.234 ^b^

^a^Independent-samples *t* test

^b^Wilcoxon-Mann–Whitney test

^c^Fisher’s exact test

**Table 6:** Operative space on the midbrain surface of the group nl related with lesion size and mRS at follow-up

	Distance between MCM and midbrain surface (median: 3 mm, IQR: 2–4 mm)			Width of midbrain aperture (median: 5 mm, IQR: 5–7 mm)		
	≤ 3 mm	> 3 mm	*P* value	≤ 5 mm	> 5 mm	*P* value
Patients, *n*	15	8		15	8	
Lesion size (mean ± SD), mm	16.1 ± 7.32	12.9 ± 5.36	0.825 ^a^	12.7 ± 5.5	19.1 ± 7.26	0.681 ^a^
mRS at follow-up (mean ± SD)	0.67 ± 0.98	0.5 ± 0.76	0.875 ^a^	0.67 ± 0.98	0.5 ± 0.76	0.825 ^a^
mRS improved	9/15, 60%	6/8, 75%	0.657 ^b^	11/15, 73.3%	4/8, 50%	0.371 ^b^

^a^Wilcoxon-Mann–Whitney test

^b^Fisher’s exact test

### Predicting the aspect of the midbrain surface by evaluating pertinent MRI slices

As shown in Table 7, there was a discrepancy in allocating the MCMs to one of the 4 groups (*nl*, *yw*, *bg*, and *ex*) when assessing only the MRI scans on the one hand or when evaluating each case by viewing the actual video record on the other hand. Cohen’s kappa value was 0.352 (95% CI, 0.13–0.58; *p* = 0.002). The MRI appearance suggested more frequently a bulging midbrain surface than was actually found in surgery.

**Table 7:** Categorization of lesions on surgical video display and based only on the MRI appearance

Patients; groups	Video	MRI
Total	76	75
*nl*	23	16
*yw*	33	26
*bg*	14	28
*ex*	6	5

## Discussion

### Background and aim of this study

In the early period of neurosurgery, the brainstem was regarded over decades as surgical “no man’s land.” Only since the 1980s, neurosurgeons began to operate on intrinsic brainstem lesions with increasing frequency [3]. During the following years, the indication for surgery in brainstem cavernous malformations was well established, and many authors reported promising long-term results with acceptable morbidity [1, 3, 12, 22]. Most of the time, though, only cavernous malformations that reached the brainstem surface were primarily considered good candidates for surgery [1, 10, 26, 32]. Conversely, many neurosurgeons tended to maintain a cautious attitude and were thus rather reluctant to recommend surgery in intrinsic focal lesions located at some distance from the brainstem surface [1, 22]. Quite understandably, splitting the compact healthy brainstem parenchyma to expose an intrinsic deep-seated cavernous malformation carries the risk of injuring adjacent long-tract fibers and intrinsic brainstem nuclei. This is also the reason why several anatomical studies were conducted to define so-called “safe entry zones” into the brainstem [13, 14, 31].

In spite of this comprehensible reasoning, we have evacuated microsurgically during the past 2 decades a substantial number of cavernous malformations of the midbrain, pons, and medulla that were not readily visible on the surface of the brainstem, and we still have achieved most satisfactory results in these cases [4, 18, 29]. Based on these observations, we gradually learned that limited splitting of the healthy brainstem parenchyma and gentle microsurgical manipulation within the brainstem were tolerated well and, with few exceptions, did not cause additional or at least no permanent neurological long-term sequelae. During each surgical procedure, we kept the long sensory and motor tracts, the auditory pathways, as well as the integrity of cranial nerves III and IV under rigorous surveillance. We gained experience with continuous electrophysiological monitoring over several decades, and accordingly, we can clearly estimate its importance in guiding the surgeon during the entire procedure and particularly during microsurgical intrinsic brainstem manipulation. On the other hand, we must mention that despite its recognized value, electrophysiological monitoring was certainly not the only decisive factor for a good outcome, not even in *nl* lesions.

We found only one previous study that mentioned a better outcome in patients with deep lesions compared to those with a lesion of moderate depth [7]; this is a valuable study despite some limitations: the number of patients was rather small (the midbrain was involved in only 11 patients), and surgery was indicated only when the deep lesion appeared reachable via a safe entry zone into the brainstem; moreover, the terms “deep” and “moderate depth” were not defined by exact measurements.

The goal of the present study was to verify the impact of MCM depth on the surgical outcome after lesionectomy. For a systematic analysis in our quite large patient series, it seemed expedient to divide all MCMs according to their exact depth within the midbrain and based on how they had altered the brainstem surface. To our knowledge, this is the first study in a larger patient population that sheds more light onto this important aspect.

### Surgical access and entry points into the midbrain

Ideally, all portions of an intrinsic MCM should be readily accessible at surgery. Choosing the optimal surgical approach plays therefore a crucial role in the surgical management.

The midbrain can be exposed practically in a 360-degree fashion. In the present series, we approached the lesions from anteriorly via the anterior interhemispheric approach, from anterolaterally using a pterional or orbitozygomatic craniotomy and transsylvian exposure, from laterally by applying the subtemporal approach, from posterolaterally via the lateral supracerebellar infratentorial approach, and from posteriorly by utilizing the median supracerebellar infratentorial or transtentorial approach. Each surgical approach provided a “surgical window” that gave access to a certain, albeit limited, area of the midbrain. Anticipating the extent and limits of this surgical window was crucial for safely removing the MCMs. To choose the optimal approach, we took into consideration several morphological features: lesion size and shape (oval vs. spheric), the exact location within the midbrain (peduncle, tegmentum, tectum), its three-directional extension as described in our previous classification [29], and last but not least, the expected relationship between MCM and midbrain surface as described in this study. Moreover, the vascular anatomy of the midbrain surface in the area overlying the MCM also played an important role during surgery when it came to choose the exact entry point into the midbrain.

As other neurosurgeons, we tended to expose the MCMs in the area where we expected the lesion to be closest to the midbrain surface or where it seemed to abut the pial surface [4, 13, 14, 21]. The choice of the exact entry point into the brainstem (see Fig. 6) was either predetermined by the lesion (in group *ex* MCMs) or rather straightforward in MCMs of the group *bg*. Selecting the exact entry point into the midbrain in *nl* or in *yw* lesions was far more demanding because neither hemosiderin discoloration nor a prominent midbrain surface facilitated the choice of the entry point in these patients that comprised 74% of all individuals. In these cases, we had to rely to a great extent on specific anatomical knowledge and the presumed intrinsic midbrain morphology derived from preoperative MRIs [9, 21, 23, 31]. Even though guidance by neuronavigation was helpful in several instances, we never relied entirely on this technical tool because of possible inaccuracies. Another important issue in choosing an optimal entry point into the midbrain was our constant attempt to keep the midbrain aperture as little as possible, while still exposing all MCM portions in the limited surgical cavity without significantly compromising the midbrain. We punctured the midbrain with the bipolar forceps and gently dilated the superficial parenchymal layer as exemplified in Figs. 2e and 3e. To avoid leaving MCM remnants behind and thus prevent recurrent hemorrhage, our microsurgical efforts also concentrated on complete lesionectomy.

**Fig. 7:**
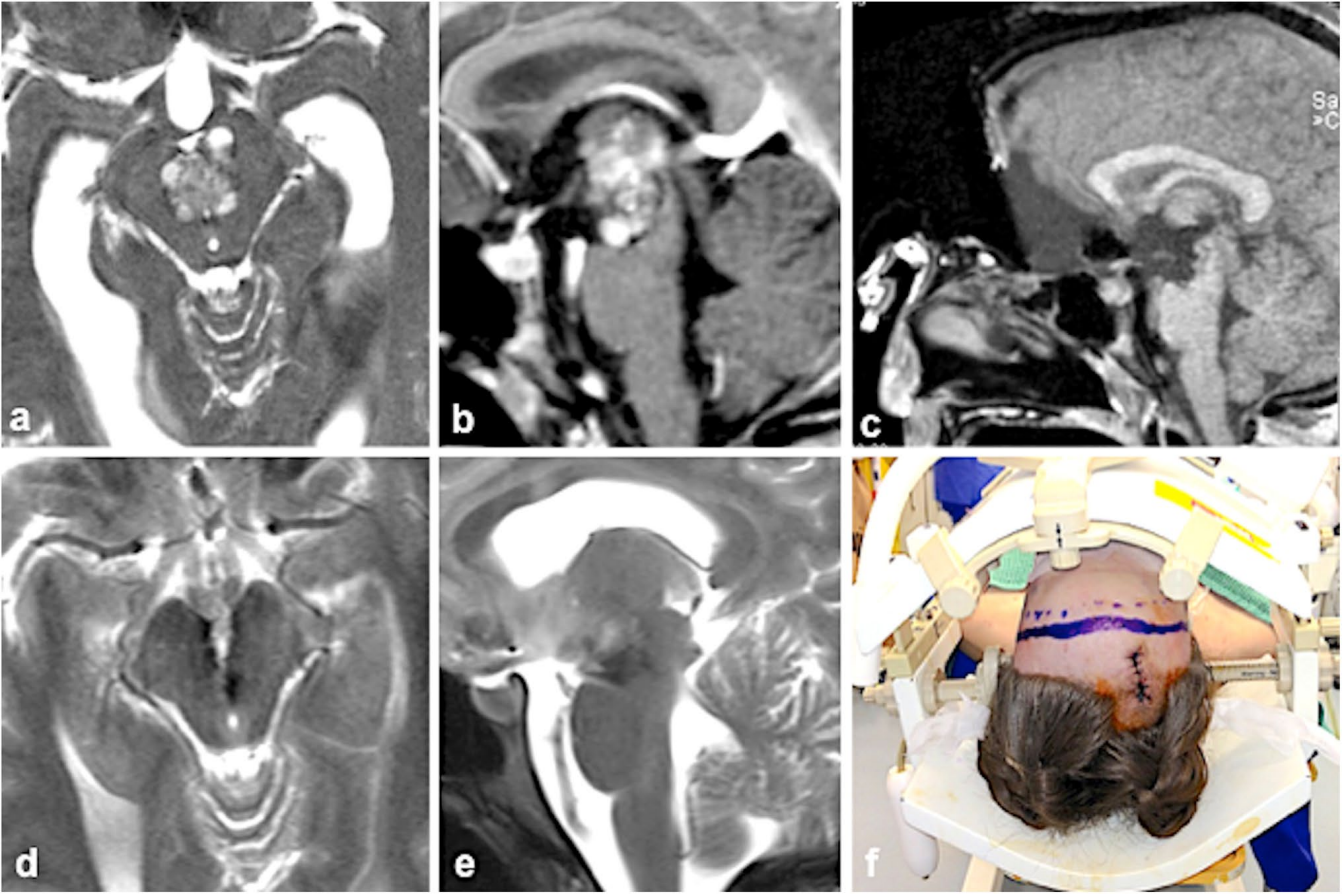
This 21-year-old woman presented with transient oculomotor nerve palsy; the MRI revealed a centrally located MCM (**a**) with thalamic extension whose upper part reached the floor of the third ventricle (**b**). Due to diplopia, severe headache, and vomiting caused by obstructive hydrocephalus, she underwent endoscopic third-ventricle ventriculostomy. Several days later, surgical MCM resection was undertaken via the anterior interhemispheric approach; the sagittal intraoperative MRI demonstrates complete lesionectomy (**c**); axial (**d**) and sagittal post-surgical MRI also confirmed complete MCM resection (**e**). The head was fixed in a special headrest with MRI coils; a coronal incision is marked on the skin (**f**). Postoperatively, slightly accentuated oculomotor nerve palsy was present that normalized within 3 months

**Fig. 8:**
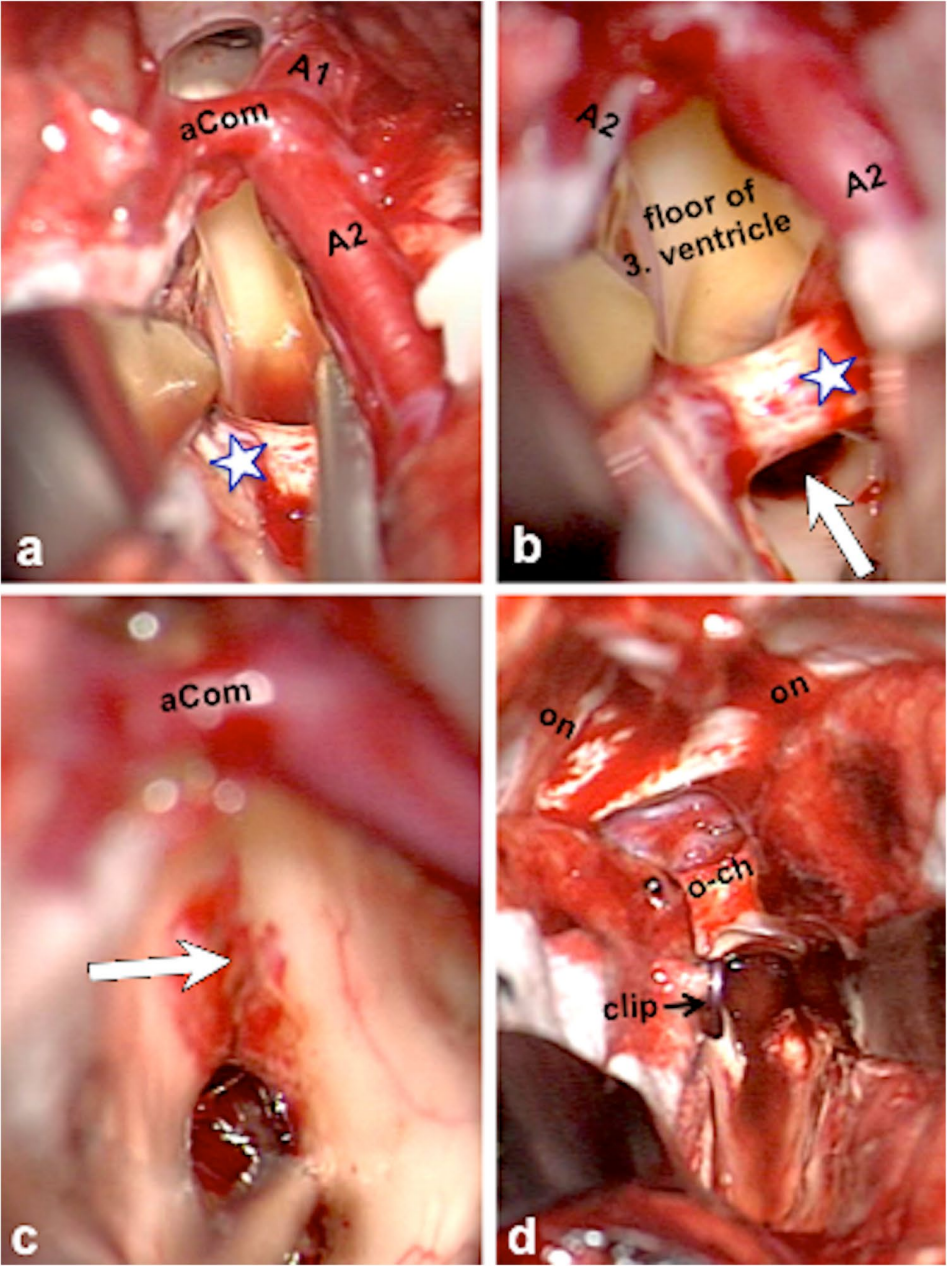
Intraoperative photographs of the patient whose case is shown in Fig. 7. Via the frontobasal interhemispheric access route, the anterior circle of Willis (*A1*, *aCom*, and *A2*) was exposed (**a**); the lamina terminalis was opened superiorly beyond the level of the anterior commissure (*asterisk*), giving a wide access to the floor of the third ventricle (**b**); the arrow indicates the area where the underlying MCM was initially exposed. The integrity of the anterior commissure (*asterisk*) was kept intact throughout the procedure. To reach the caudal MCM portions, the floor of the third ventricle (*arrow*) was split towards anteriorly (**c**). Sufficient working space was achieved only by subsequently clipping the anterior communicating artery (*aCom*). Using two self-retaining retractors, a wide exposure of the anterior frontal base was obtained (**d**) including both olfactory nerves (*on*) detached from the frontal lobe and the optic chiasm (*o–ch*); two small Aesculap aneurysm clips were used to interrupt the anterior communicating artery that was divided between these clips (*clip*)

In MCMs located anteriorly and in the center of the midbrain, we have chosen the anterior midline approach as exemplified in Figs. 7 and 8. This infrequently utilized surgical exposure was laborious and required a profound anatomical knowledge of the optico-hypothalamic area and anterior aspect of the midbrain [14]. In some patients, we preferred to divide the anterior communicating artery to obtain safe access to the interpeduncular area and to the anterior third ventricle as we have described in more detail elsewhere [28] and as we have applied in the patient shown in Figs. 7 and 8.

**Fig. 9:**
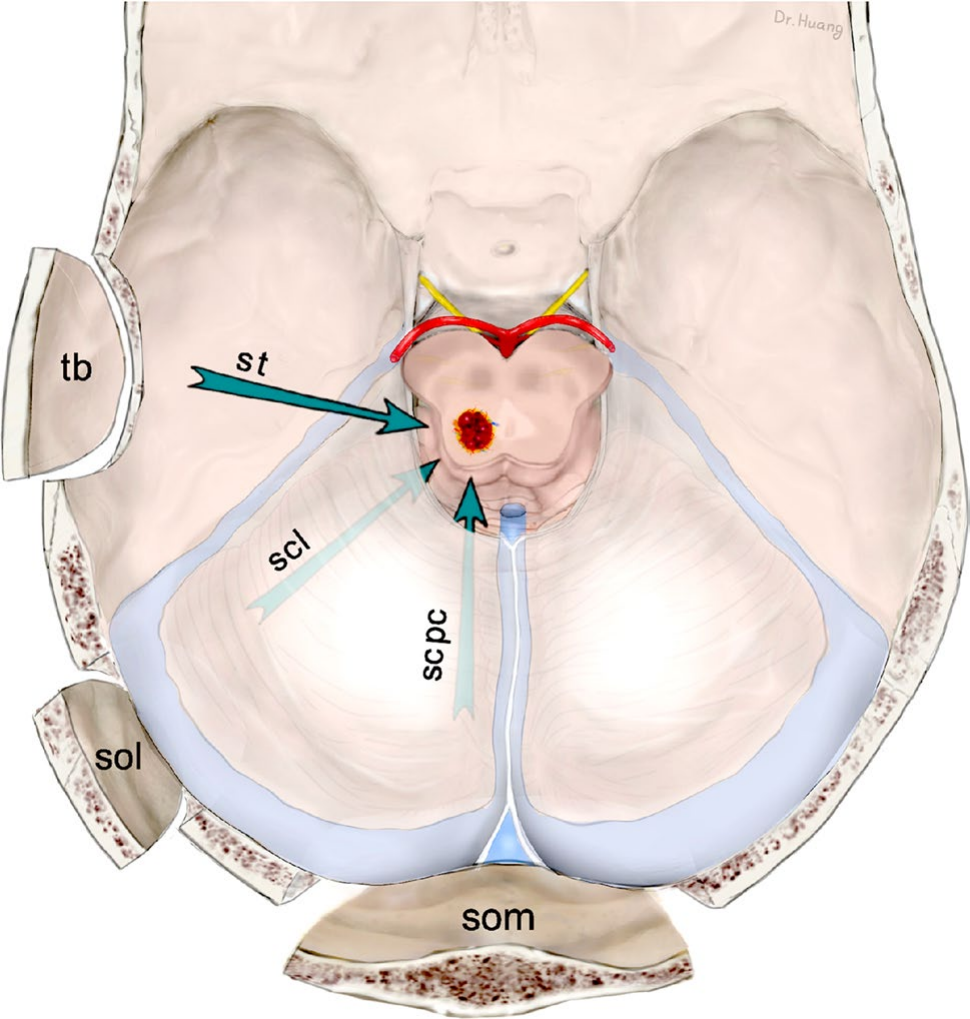
Artistic illustration showing a deep-seated MCM in the right tectal/tegmental region. This lesion may be exposed from 3 directions using 3 different craniotomies and three different access routes as indicated by the green arrows. tb, temporobasal (craniotomy); sol, suboccipital lateral; som, suboccipital medial; st, subtemporal (approach); scl, supracerebellar infratentorial lateral; scpc, supracerebellar infratentorial paraculminal

In some instances, the MCM appeared accessible from 2 or even 3 different viewing angles, each requiring a different craniotomy and a distinct surgical access route as illustrated in Fig. 9. In such cases, more than one surgical approach was considered a possible and valid option. The final decision, however, either depended on the craniocaudal MCM extension or was undertaken according to the longitudinal axis in oval-shaped lesions.

**Fig. 10:**
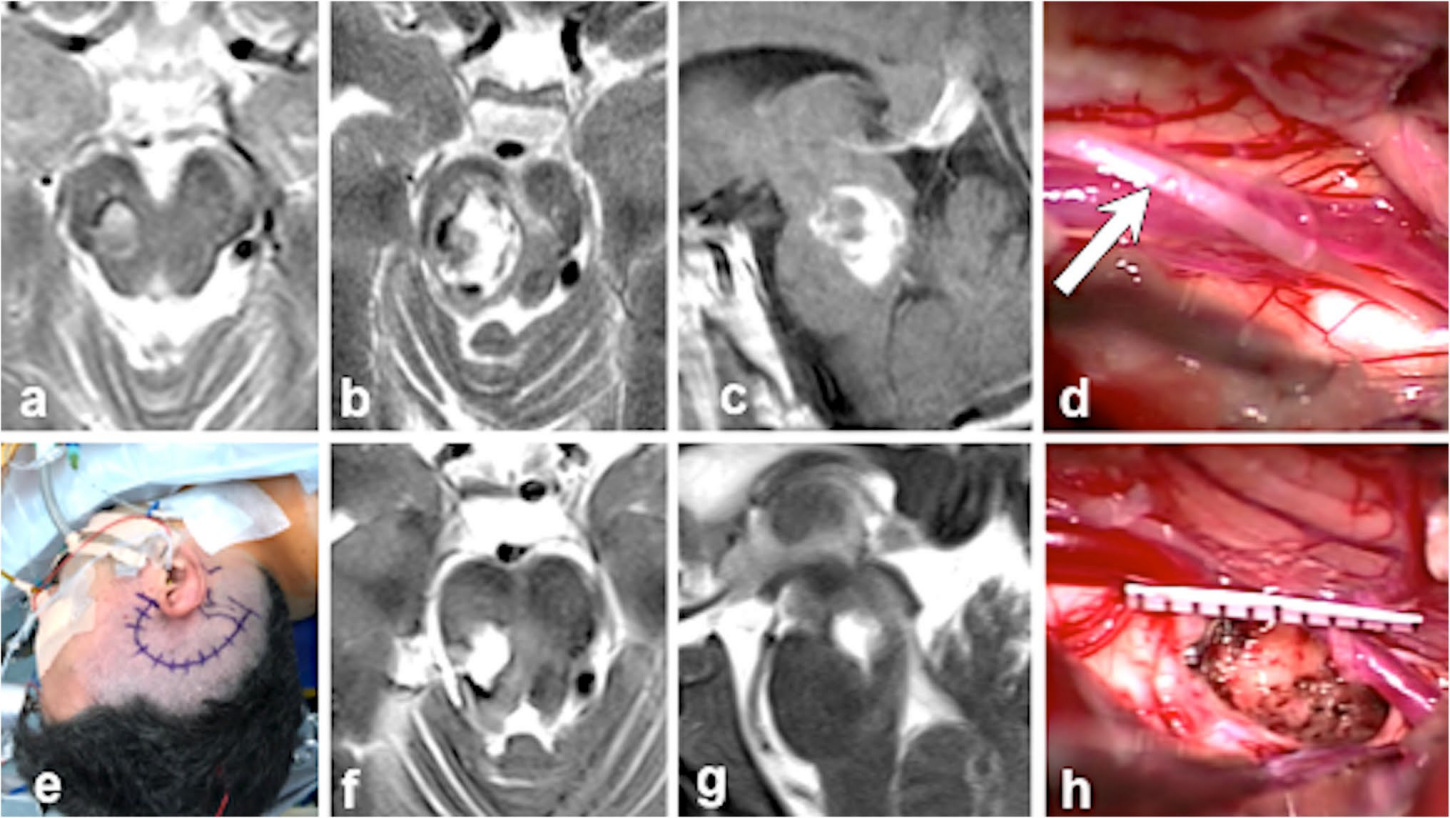
This 36-year-old male harbored multiple cavernous malformations in the basal ganglia, cerebellum, and midbrain. Three years prior to surgery, he suffered the first brainstem hemorrhage, fortunately without neurological deficit. Six months before surgery, a slight hemorrhage was noted on MRI (**a**), but the patient was asymptomatic by that time; 3 weeks before admission, a new bleeding episode occurred that caused left-sided hemiparesis and hemihypesthesia, right sixth and seventh nerve palsy, and deteriorated consciousness. Preoperative axial (**b**) and sagittal MRI showed a 22-mm hemorrhagic MCM extending caudally (**c**). He underwent surgery via the right subtemporal approach; intraoperatively, the surface of the tegmentum was prominent at the level of the fourth cranial nerve (*arrow*, **d**). Surgery was performed with the patient in the supine position (**e**). Postoperative axial (**f**) and sagittal (**g**) MRI as well as the intraoperative photograph (**h**) confirmed complete MCM removal. The patient rapidly recovered after surgery, and after 6 months, he remained without permanent neurological deficits

Generally, lesions with substantial caudal extension into the pons were rather exposed via the subtemporal access route [3, 17, 25, 27]. We used to enlarge the surgical window by dividing the tentorium as exemplified in Fig. 10. Diffusion tensor imaging and tractography are useful in visualizing the corticospinal and sensory tracts [5, 11, 15, 16, 27, 30], but its true practical value for MCM removal was so far rather limited in our experience.

**Fig. 11:**
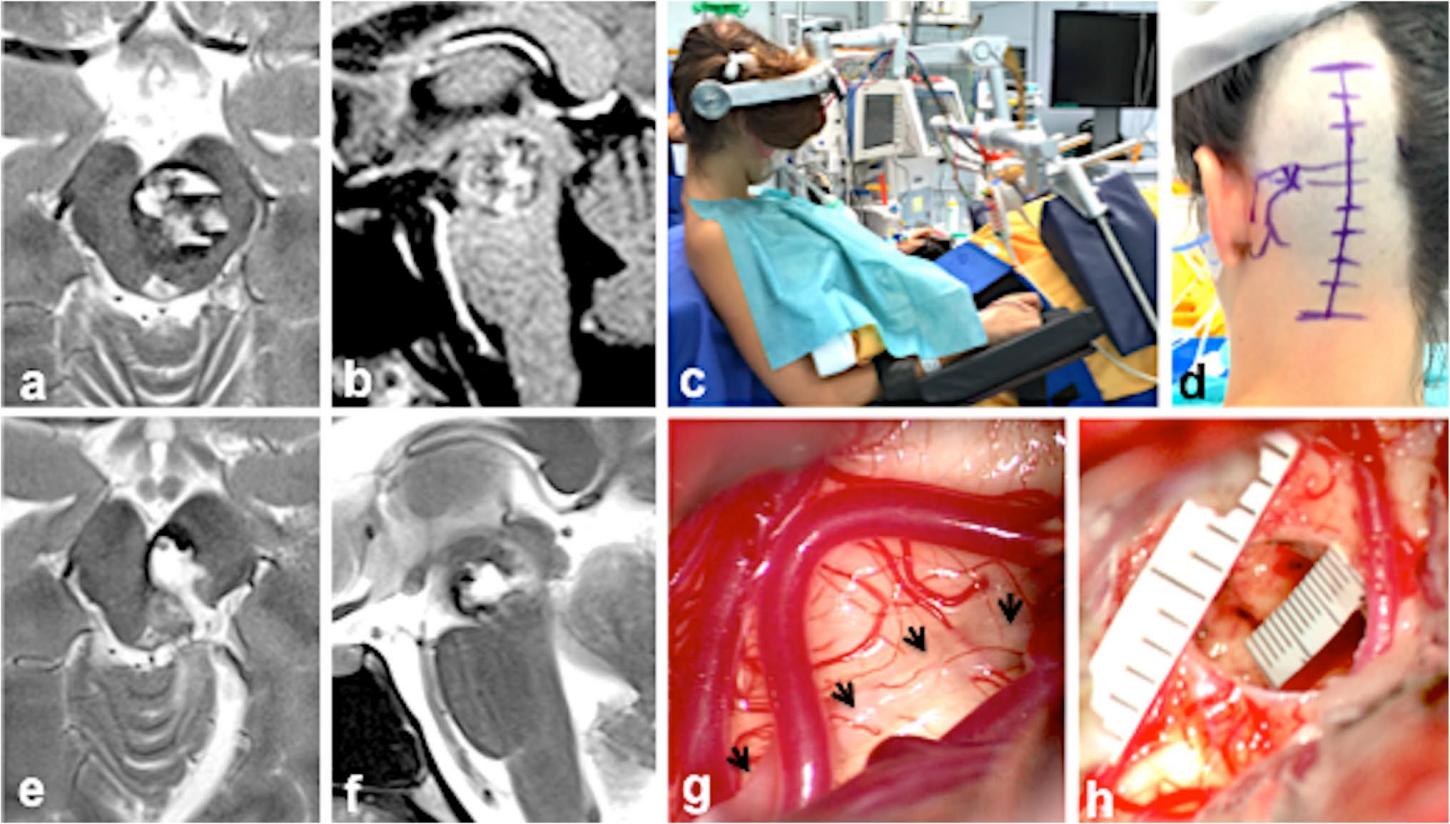
This 19-year-old female presented with a history of two repeated hemorrhages from an intra-axial MCM. Her symptoms consisted of headache, diplopia, and slight motor weakness of her right-sided body. Preoperative axial (**a**) and sagittal (**b**) MRI showed a 20-mm-sized type *nl* MCM in the tegmentum region. She underwent surgery in the semi-sitting position (**c**) via a lateral supracerebellar transtentorial approach; a left-sided lateral occipital/suboccipital longitudinal incision was marked on skin (**d**). Postoperative axial (**e**) and sagittal (**f**) MRI confirmed total lesionectomy. At surgical exposure, the lateral surface of the tegmentum appeared normal; the arrowheads indicate the course of the trochlear nerve (**g**). The intraoperative photograph taken after complete MCM removal shows the empty resection cavity with two millimetre  scales (**h**). Postoperatively, a left-sided partial oculomotor paresis was present that gradually improved over the following weeks up to normal function. No other neurological deficits were noted

Posterior or posterolateral exposures were preferred more often in MCMs with superior extension into the thalamus. The lateral supracerebellar infratentorial approach (Fig. 11) is most useful to reach the posterolateral aspect of the midbrain [2, 6, 9, 13, 19, 24] and offers a straight-line viewing trajectory to the lateral mesencephalic sulcus that is regarded as one of the safe entry zones into the midbrain. In rare cases, a midbrain MCM can even be exposed through the fourth ventricle using a telovelar exposure of the rhomboid fossa and a transaqueductal access route [5, 8, 20].

**Fig. 12:**
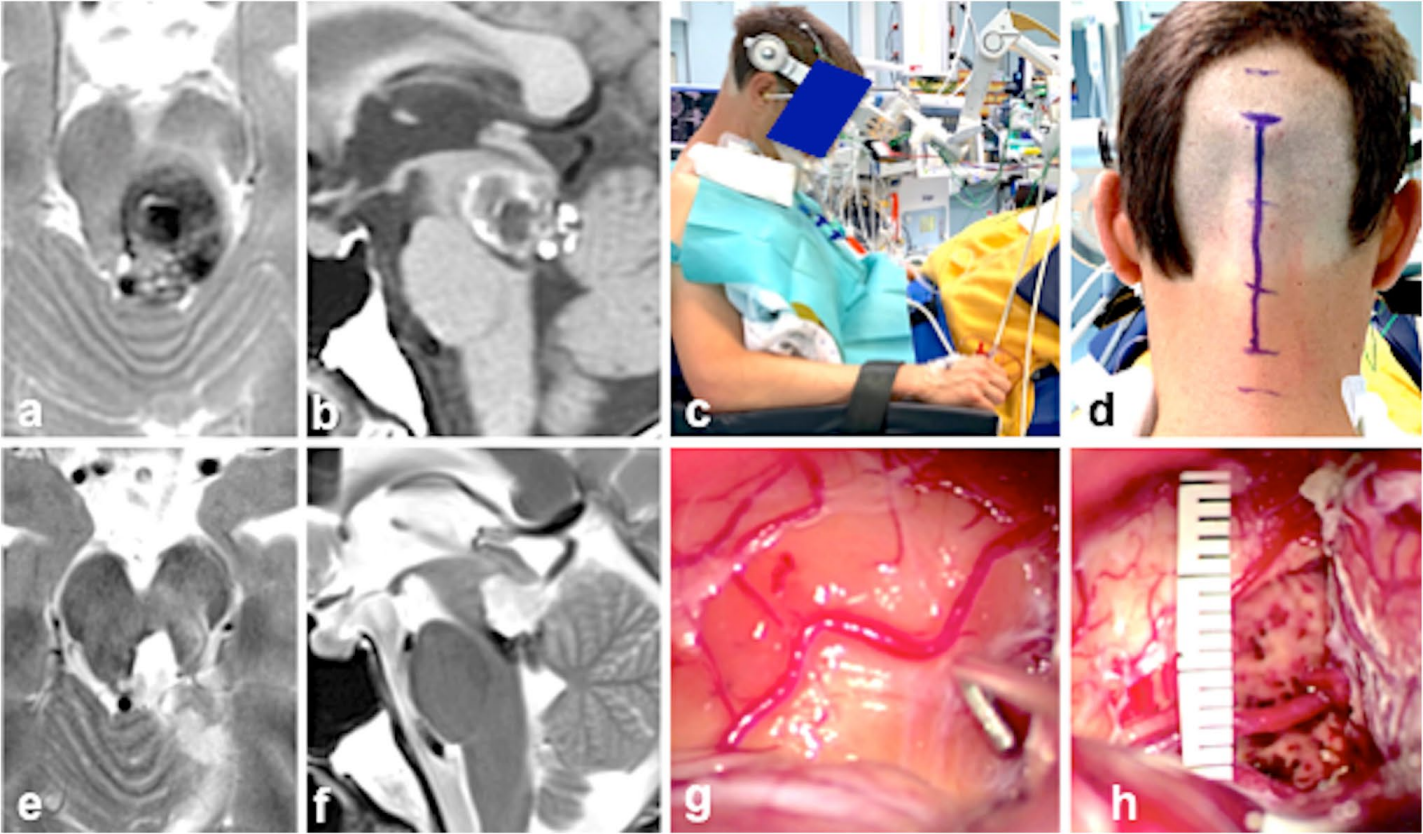
Over 5 years, this 35-year-old male suffered from recurrent headache and diplopia. Three months before surgery, the eye movement disorder worsened, and additional progressive gait instability was noted; the symptoms were caused by midbrain hemorrhage that gradually increased to a remarkable size as seen on preoperative axial (**a**) and sagittal (**b**) MRI. The patient underwent surgery in the semi-sitting position with the head flexed (**c**); a longitudinal midline occipital/suboccipital incision was marked on the skin (**d**). Postoperative axial (**e**) and sagittal (**f**) MRI confirmed total MCM resection. At surgery, the surface of the right midbrain tectum was prominent, but the underlying MCM was still covered by a thin layer of parenchyma (**g**). Total lesionectomy was also confirmed in surgery; the pial midbrain aperture measured 12 mm (**h**)

We exposed most of the tectal MCMs in this series via the supracerebellar infratentorial paraculminal exposure (Fig. 12). This approach is facilitated by extending the craniotomy superiorly beyond the level of the transverse sinus and necessitates the preservation of supracerebellar bridging veins [2].

### Interpretation of the results

As shown in our previous publication, 90% of MCM patients were in good or excellent clinical condition by the time of follow-up (mRS of 0–2) [29].

In the present study, we placed the emphasis on a different aspect. We are not aware about any previous publication that has systematically dealt with the impact of MCM depth on the surgical outcome. Also, for the first time, we have divided MCMs into the 4 distinct categories described in the “Results” section. Understandably, our main focus lay on lesions of the *nl* group because of their deep intrinsic location and the apparently normal midbrain surface. Contrary to any empiric expectation, we found that the surgical long-term outcome in *nl* patients was not worse but even better than in the groups *bg* and *ex* and in the entire patient population. This result demonstrates that a deep intrinsic location per se is not an unfavorable feature. We must admit, though, that the MCMs in the group *nl* were relatively small in size and, except for one case, mostly with good mRS on admission. Also, some of these lesions contained intralesional hematomas that already had expanded the midbrain to some degree. Once these encapsulated hematomas were evacuated surgically, additional working space became available that facilitated microsurgical manipulation and subsequent MCM resection. This might be another reason for the favorable surgical outcome in these cases.

In the same context, we examined several factors in the group *nl* that potentially could predict the outcome. Obviously, our results did not confirm the abovementioned empirical assumption; instead, we found that none of the examined factors, in particular neither lesion size nor MCM depth or size of the midbrain aperture, turned out as predictors of outcome.

### Rating the superficial aspect of the midbrain on preoperative MRI scans

The possibility of predicting the aspect of the midbrain surface by evaluating pertinent preoperative MRI scans might be advantageous to plan the surgical approach and to locate a suitable entry point into the brainstem. In this context, we were interested in determining the reliability of preoperative MRI evaluation. While T1-weighted MRI sequences usually did not show the entire hemosiderin-loaded gliotic tissue that surrounded the MCM, T2-weighted sequences tended to slightly exaggerate the lesion’s circumferential contour. When we compared the superficial midbrain aspect as predicted by MRI scans with the intraoperative appearance on respective video clips, for instance in the patient shown in Fig. 12, the MRI scans more often suggested a discolored and bulging midbrain surface than it was actually the case in surgery. Our statistical analysis confirmed that evaluating preoperative MR images alone was not sufficiently reliable in predicting the exact aspect of the midbrain surface that overlay the intrinsic MCM.

## Conclusions

For the first time, this study demonstrates in a substantial patient population that a deep intrinsic location of MCMs is not necessarily associated with an unfavorable clinical outcome after microsurgical lesionectomy as might have been expected empirically. The surgical outcome in our patient group *nl* was even superior to the result in the groups *bg* and *ex* and in the entire patient population. Neither lesion size nor width of midbrain aperture or distance between MCM and midbrain surface correlated with the outcome in the *nl* patient group. Predicting the aspect of the midbrain surface by evaluating preoperative MR images alone was not sufficiently reliable. The MRI aspect suggested more frequently a bulging midbrain surface than it was actually found in surgery.

## Abbreviations

CM: Cavernous malformation
IQR: Interquartile range
MCM: Midbrain cavernous malformation
mRS: Modified Rankin Scale Score
